# Supplementary figures and images for: Loss of the Aspergillus fumigatus spindle assembly checkpoint components, SldA or SldB, generates triazole heteroresistant conidial populations
Source: Microbiol Spectr. 2025 Jun 16;13(8):e00536-25. doi: 10.1128/spectrum.00536-25 (PMC12323316; doi:10.1128/spectrum.00536-25)

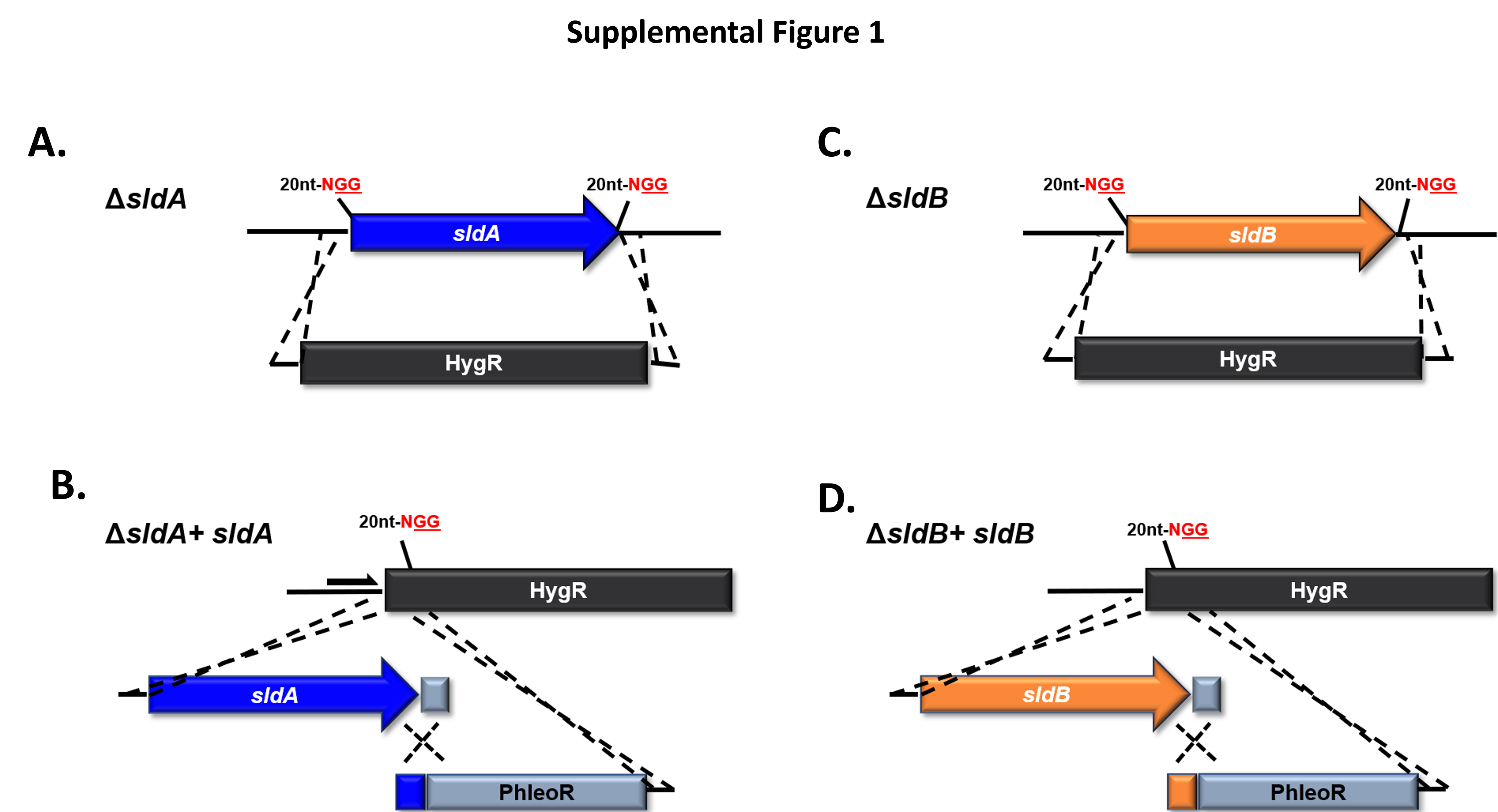

Supplement: Fig. S1 — Schematics for gene targeting. [file spectrum.00536-25-s0001.tif]

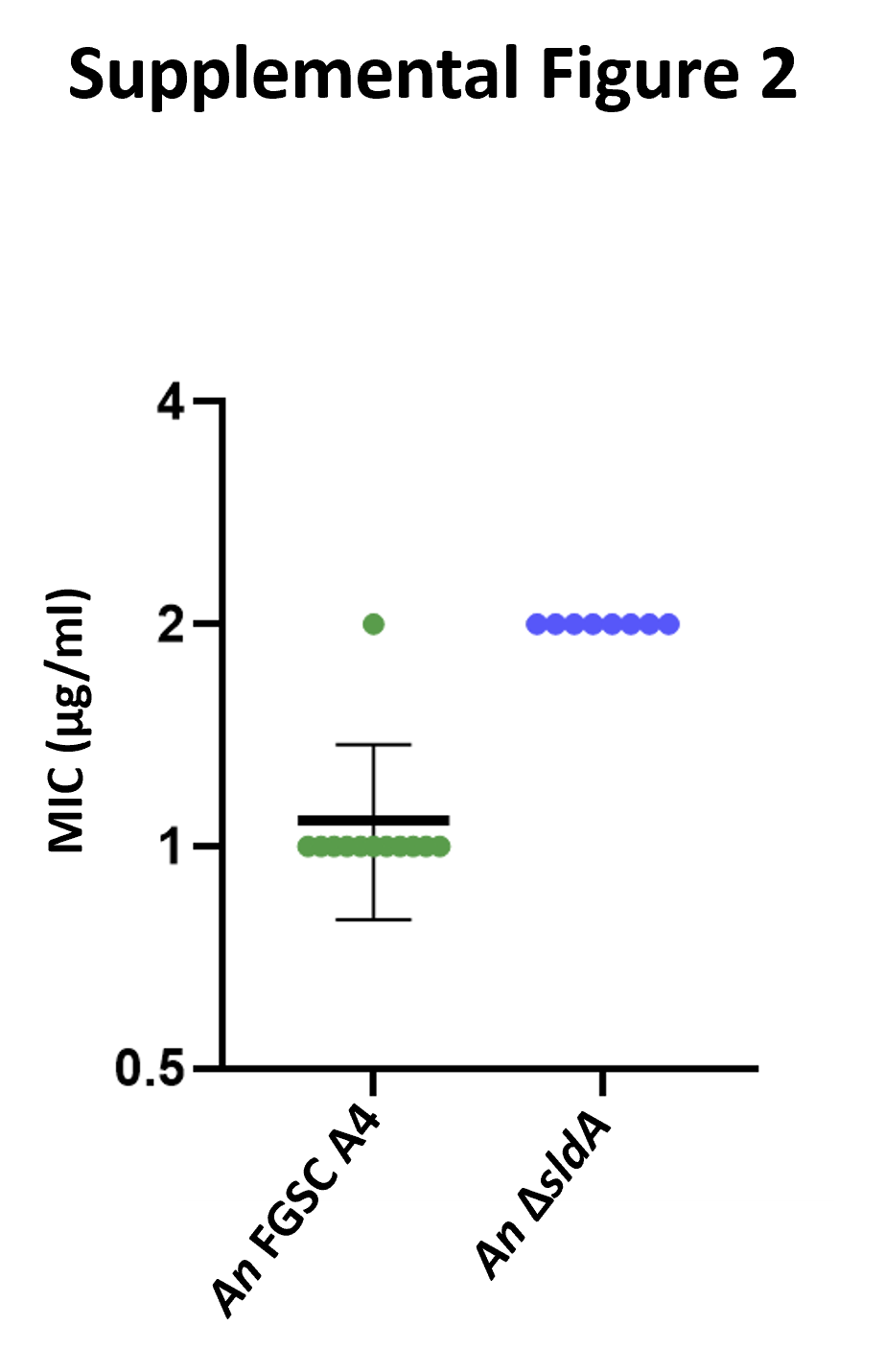

Supplement: Fig. S2 — Triazole MIC of Aspergillus nidulans sldA mutant. [file spectrum.00536-25-s0002.tif]

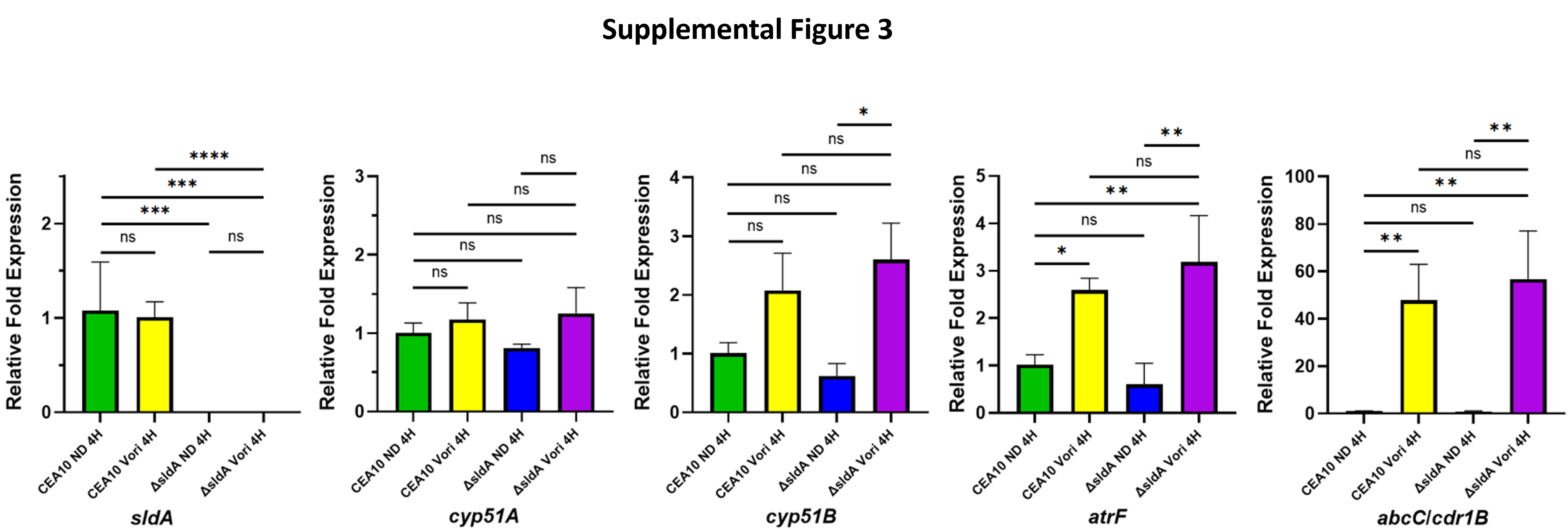

Supplement: Fig. S3 — Gene expression analyses. [file spectrum.00536-25-s0003.tif]
