# Supplementary material for: Loss of the Aspergillus fumigatus spindle assembly checkpoint components, SldA or SldB, generates triazole heteroresistant conidial populations
Source: Microbiol Spectr. 2025 Jun 16;13(8):e00536-25. doi: 10.1128/spectrum.00536-25 (PMC12323316; doi:10.1128/spectrum.00536-25)
Supplement: Supplemental material — Legends for all Supplemental figures. [file spectrum.00536-25-s0004.docx]

**Supplemental Figure 1. Schematic for construction of gene deletion mutants. A)** Schematic of *sldA* gene deletion by CRISPR-Cas9-mediated excision and subsequent replacement with a PCR-amplified homologous repair template containing the gene encoding for resistance to hygromycin. **B)** Schematic of *sldA* gene complementation by CRISPR-mediated re-insertion of the entire ORF into the genome at the native locus joined with a homologous repair template containing the gene encoding resistance to phleomycin. **C)** Schematic of *sldB* gene deletion by CRISPR-Cas9-mediated excision and subsequent replacement with a PCR-amplified homologous repair template containing the gene encoding for resistance to hygromycin. **D)** Schematic of *sldB* gene complementation by CRISPR-mediated re-insertion of the entire ORF into the genome at the native locus joined with a homologous repair template containing the gene encoding resistance to phleomycin.

**Supplemental Figure 2. The role of *sldA* in triazole susceptibility is conserved in *A. nidulans*.** The MIC to voriconazole was determined by broth microdilution assay in GMM supplemented with 0.5% yeast extract (GMM+YE) after 48h at 37°C. Individual results from multiple experiments are shown within the graph. Bold central line denotes the Mean MIC. Error bars represent SD.

**Supplemental Figure 3. Loss of *sldA* does not result in differential expression of genes commonly associated with triazole resistance.** Analysis for differential expression of resistance-associated genes by RTq-PCR. Strains were assessed for differences in expression at baseline and upon exposure to sub-inhibitory level of voriconazole (0.50 µg/ml). Expression was analyzed after 4 hrs of incubation in the presence of drug at 37°C. Error bars represent SD. Statistical analysis by One Way ANOVA with Tukey’s post hoc test. * = Significant at P<0.05 ** = Significant at P<0.01 *** = Significant at P<0.001 **** = Significant at P<0.0001.
